# Supplementary material for: Development and characterization of a 2D porcine colonic organoid model for studying intestinal physiology and barrier function
Source: PLoS One. 2025 May 7;20(5):e0312989. doi: 10.1371/journal.pone.0312989 (PMC12057940; doi:10.1371/journal.pone.0312989)
Supplement: S1 Table — (DOCX) [file pone.0312989.s001.docx]

**S 1 Table: Composition of organoid medium**, which is used for the 3D culture

| Culture medium ingredients | manufacturer |
| --- | --- |
| Advanced DMEM/F-12 | Thermo Fisher Scientific, Waltham, USA |
| 50 % L-WRN Supernatant | Self-made as described by Miyoshi et al. [1] |
| 2 mM GlutaMAX™ Supplement | Thermo Fisher Scientific, Waltham, USA |
| 10 mM Hepes solution | Sigma-Aldrich, Schnelldorf, Germany |
| 100 U/ml penicillin  0,1 mg/ml streptomycin | Thermo Fisher Scientific, Waltham, USA |
| 100 U/ml polymyxin B sulfate salt | Sigma-Aldrich, Schnelldorf, Germany |
| B-27™ supplement (1x), serum free | Thermo Fisher Scientific, Waltham, USA |
| 50 ng/ml recombinant murine EGF | Prepotech, New Jersey, USA |
| 1 mM N-Acetyl-L-cysteine | Sigma-Aldrich, Schnelldorf, Germany |
| 10 µM Y-27632 dihydochloride | MedChemExpress, New Jersey, USA |
| 500 nM A 83-01 | MedChemExpress, New Jersey, USA |
| 10  µM SB 202190 | MedChemExpress, New Jersey, USA |
| 10 nM Gastrin I | MedChemExpress, New Jersey, USA |

L-WRN: L cell line expressing Wnt3a, R-Spondin and noggin

## References

1. Miyoshi H, Ajima R, Luo CT, Yamaguchi TP, Stappenbeck TS. Wnt5a potentiates TGF-beta signaling to promote colonic crypt regeneration after tissue injury. Science. 2012;338(6103):108-13.
