# Supplementary material for: Development and characterization of a 2D porcine colonic organoid model for studying intestinal physiology and barrier function
Source: PLoS One. 2025 May 7;20(5):e0312989. doi: 10.1371/journal.pone.0312989 (PMC12057940; doi:10.1371/journal.pone.0312989)
Supplement: S3 Table — (DOCX) [file pone.0312989.s003.docx]

**S 3 Table: Composition of Differentiation medium**

| Culture medium ingredients | manufacturer |
| --- | --- |
| Advanced DMEM | Thermo Fisher Scientific, Waltham, USA |
| 5 % L-WRN Supernatant | Self-made, as described by Miyoshi et al. [1] |
| 20 % Fetal bovine serum | Sigma-Aldrich, Schnelldorf, Germany |
| 2 mM L-glutamine | Sigma-Aldrich, Schnelldorf, Germany |
| 100 U/ml penicillin  0.1 mg/ml streptomycin | Thermo Fisher Scientific, Waltham, USA |
| 100 U/ml polymyxin B | Sigma-Aldrich, Schnelldorf, Germany |
| 50 ng/ml EGF | Prepotech, New Jersey, USA |
| 5 µM DAPT | MedChemExpress, New Jersey, USA |

L-WRN: L cell line expressing Wnt3a, R-Spondin and noggin

## References

1. Miyoshi H, Ajima R, Luo CT, Yamaguchi TP, Stappenbeck TS. Wnt5a potentiates TGF-beta signaling to promote colonic crypt regeneration after tissue injury. Science. 2012;338(6103):108-13.
