# Supplementary material for: Development and characterization of a 2D porcine colonic organoid model for studying intestinal physiology and barrier function
Source: PLoS One. 2025 May 7;20(5):e0312989. doi: 10.1371/journal.pone.0312989 (PMC12057940; doi:10.1371/journal.pone.0312989)
Supplement: S4 Table — (DOCX) [file pone.0312989.s004.docx]

**S 4 Table: SYBR Green primers for SYBR Green® assay.** If not stated otherwise the primers were self-designed

| Gene |  | Primer and probes (5‘→ 3‘) | fragment | Source |
| --- | --- | --- | --- | --- |
| CaCC | Forward | ACCAGGCTGGGTAGTGAATG | 161 bp | this study |
|  | Reverse | GTGGGTAGAGGTCAGGCAAG |  |  |
| CFTR | Forward | ATTGGAGCTGTGGCAGTTGT | 199 bp | this study |
|  | Reverse | AGGCTCGAAGGGTCCATAGT |  |  |
| CLDN 3 | Forward | TCATCGGCAGCAGCATTATC | 105 bp | [1] |
|  | Reverse | GAGAGTCGTACACTTTGCACTG |  |  |
| ENaC alpha | Forward | GTTCCAAGTACACGCAGCAG | 117 bp | this study |
|  | Reverse | AGTCACAGAACTCCATGCCC |  |  |
| ENaC beta | Forward | TGCAACGACACCCAGTACAA | 194 bp | this study |
|  | Reverse | GCTGCTGACTCCTCAATGGT |  |  |
| ENaC gamma | Forward | GTTGCCAGTCTGTGTGCAAG | 235 bp | this study |
|  | Reverse | ATCTCGATGCTATTGGCGGG |  |  |
| ENaC delta | Forward | TGGGCTTCCGACTGTGTAAC | 176 bp | this study |
|  | Reverse | GCAGGAGGAGACCAAGTGAC |  |  |
| MUC2 | Forward | TGTGTCATCAACTTGCCCGA | 120 bp | this study |
|  | Reverse | GGAGATGAGCTGGTCGTGTG |  |  |
| MUC4 | Forward | GCCTCCCAAGCAGATGTCAA | 122 bp | this study |
|  | Reverse | TTTCCTGAGGCCGGTATTGC |  |  |
| MUC5AC | Forward | AAGTAAGCCCACCTCACACG | 102 bp | this study |
|  | Reverse | TTTCTGTCTGGTCCGGGGTA |  |  |
| NHE1 | Forward | AACAACTACCTGACGGTGCC | 165 bp | this study |
|  | Reverse | TTCATTCACCAGGTCCACGG |  |  |
| NHE2 | Forward | ATTTCTAGCGCAGGTGGCAA | 160 bp | this study |
|  | Reverse | ACTCAACGTCCACCTCGTTC |  |  |
| NHE3 | Forward | AACACCTCTACAGCCGACAC | 110 bp | this study |
|  | Reverse | CTTGGCCGACTTGAAGGACT |  |  |
| OCLN | Forward | CTACTCGTCCAACGGGAAAG | 158 bp | [2] |
|  | Reverse | ACGCCTCCAAGTTACCACTG |  |  |
| ZO-1 | Forward | AGAGCCTGCCAAGTCAGTTC | 131 bp | this study |
|  | Reverse | TTGCCTGCAGTGGGTCATAG |  |  |
| RPL4 | Forward | AGCCCAGAGATCCAAAGAGC | 143 bp | this study |
|  | Reverse | CGAAGAATGGTGTTTCGGCG |  |  |

## References

1. Reddy KE, Song J, Lee HJ, Kim M, Kim DW, Jung HJ, Kim B, Lee Y, Yu D, Kim DW, Oh YK, Lee SD. Effects of High Levels of Deoxynivalenol and Zearalenone on Growth Performance, and Hematological and Immunological Parameters in Pigs. Toxins (Basel). 2018;10(3).

2. Chen H, Mao X, He J, Yu B, Huang Z, Yu J, Zheng P, Chen D. Dietary fibre affects intestinal mucosal barrier function and regulates intestinal bacteria in weaning piglets. Br J Nutr. 2013;110(10):1837-48.
