# Supplementary material for: Development and characterization of a 2D porcine colonic organoid model for studying intestinal physiology and barrier function
Source: PLoS One. 2025 May 7;20(5):e0312989. doi: 10.1371/journal.pone.0312989 (PMC12057940; doi:10.1371/journal.pone.0312989)
Supplement: S5 Table — (DOCX) [file pone.0312989.s005.docx]

**S 5 Table: TaqMan primers for TaqMan® assay**

| Gene |  | Primer and probes (5‘ → 3‘) | fragment | Source |
| --- | --- | --- | --- | --- |
| CLDN 2 | Forward | CCAAAGACAGAGTGGCGGT | 196 bp | [1] |
|  | Reverse | TCAAATTTCATGCTGTCAGGCAC |  |  |
|  | probe | TCCTGGGCTTCATCCCYGTTGC |  |  |
| Sodium glucose cotransporter | Forward | GCTTTGAATGGAATGCTCTGATT | 87 bp | [2] |
|  | Reverse | GCATCGTCACCACCCCTG |  |  |
|  | probe | AATGGGGACAAACAGCCAGCC |  |  |
| RPS18 | Forward | TGCTATCACTGCGATTAAGGGTGTA | 173 bp | [2] |
|  | Reverse | GCATAATGGTGATTACACGTTCCA |  |  |
|  | probe | ATCGACCTCACCAAGAGGGCAGG |  |  |

## References

1. Elfers K, Marr I, Wilkens MR, Breves G, Langeheine M, Brehm R, Muscher-Banse AS. Expression of Tight Junction Proteins and Cadherin 17 in the Small Intestine of Young Goats Offered a Reduced N and/or Ca Diet. PLoS One. 2016;11(4):e0154311.

2. Klinger S, Lange P, Brandt E, Hustedt K, Schroder B, Breves G, Herrmann J. Degree of SGLT1 phosphorylation is associated with but does not determine segment-specific glucose transport features in the porcine small intestines. Physiol Rep. 2018;6(1).
