# Supplementary material for: Development and characterization of a 2D porcine colonic organoid model for studying intestinal physiology and barrier function
Source: PLoS One. 2025 May 7;20(5):e0312989. doi: 10.1371/journal.pone.0312989 (PMC12057940; doi:10.1371/journal.pone.0312989)
Supplement: Attachment — Submitted filename: 20241220_Response to Reviewers.docx [file pone.0312989.s008.docx]

**Additional Editor Comments:**

Editor's comments to authors:

- The authors thank the editors for their efforts in summarizing the reviewers' feedback on our manuscript. We have addressed all individual points directly in our responses to the reviewers' comments below.

1) The limited sample size (n=2 pigs) is a major concern and has significant implications on the interpretation of the data. The manuscript should explicitly acknowledge this limitation. Including individual data points in the figures, rather than bar charts, will also improve transparency and help readers better understand data variability. The Methods section, while thorough, contains redundancies that can be streamlined for readability. For instance, routine steps such as RNA extraction need not be described in detail if they follow standard protocols. Clarity is also needed regarding native tissue usage, specifically, whether the samples were derived from the same animals or archived. Additionally, ensure the number of technical replicates is clearly stated, and define all acronyms (e.g., Isc, Rt) upon their first use.

- We acknowledge that the use of organoids from two pigs is a limitation and have explicitly addressed this in the manuscript. Each biological replicate corresponds to a separate passage of 3D organoids used to create independent 2D cultures. For gene expression, RNA was isolated from four independent 2D cultures, each with two technical replicates, while transport studies involved four independent experiments with three technical replicates each. These details, along with updated figure legends showing individual data points, provide greater transparency and help readers assess variability. We have streamlined the Methods section by removing redundant details (e.g., RNA extraction protocols) and clarified that native tissue samples were derived from four different animals. Acronyms (e.g., Isc, Rt) are now defined upon first use.

2) The Results section would benefit from adjustments to improve interpretability. For example, Figure 1 should display absolute TEER values rather than percentages to allow for meaningful comparisons with other studies. Similarly, all figures should include individual data points to reflect variability more effectively. Any missing details, such as error bars for ZO-1 gene expression on day 9 in Figure 4, should be added. Enhancing figure legends, such as clarifying aldosterone concentrations in Figure 7, will make the data presentation more complete and accessible. These are examples and the authors should refer to the individual Reviewer comments for further details.

- Thank you for your suggestions to enhance the interpretability of the Results section. We have updated all figures to include individual data points to better reflect variability. Regarding Figure 1, we chose to report relative TEER values normalized to day 8 (100%) to emphasize relative changes in barrier integrity across time points. To provide additional clarity, the mean absolute TEER value on day 8 has been included in the figure legend, facilitating comparisons with other studies.

3) A key point raised by the reviewers concerns the lack of response to carbachol in the Ussing chamber experiments, which contrasts with its reported effects in native tissue. If this discrepancy stems from methodological differences, dosing, or tissue-specific factors, these should be thoroughly discussed. While including native tissue in Ussing chamber experiments for direct functional comparison would have been ideal, if this was not feasible, the manuscript should acknowledge and explain this omission.

- Thank you for your comment regarding the carbachol response. We acknowledge that the lack of response in our study is not due to Ussing chamber sensitivity, as similar carbachol concentrations have been used successfully in other studies. We believe the discrepancy may be due to post-translational modifications affecting transporter functionality, despite comparable gene expression to in vivo conditions.

Although including native tissue for direct comparison would have been ideal, it was not included in this study. The functional physiology of native tissue in Ussing chambers has been well-established (e.g. . Leonhard-Marek et al. (1), Klinger et al. (3), Bridges et al. (4), Inagaki et al. (5)), so a direct comparison was not necessary for the scope of this work. We have revised the manuscript to clarify these points.

4) The Discussion section could benefit from a more concise and focused structure. Beginning with a brief summary of the main findings will provide a clear context for readers. Consider removing subheadings and ensuring smooth transitions between topics, such as mucin gene expression, TEER, and other findings. Expanding on functional differences and limitations, including the small sample size, lack of long-term data, and the absence of structural protein analysis will strengthen the discussion and provide valuable context for the study’s conclusions. If data on the long-term stability of the 2D cultures are not available, discussing potential implications and future directions for longer-term studies will still enhance the impact of your work.

- Thank you for your constructive feedback. We have revised the Discussion section to make it more concise and focused, starting with a brief summary of the main findings to provide clear context for readers. We have also removed subheadings and ensured smoother transitions between topics, such as mucin gene expression, TEER, and other findings. We hope these revisions improve the clarity and impact of the Discussion section.

Reviewers' comments:

5. Review Comments to the Author

Please use the space provided to explain your answers to the questions above. You may also include additional comments for the author, including concerns about dual publication, research ethics, or publication ethics. (Please upload your review as an attachment if it exceeds 20,000 characters)

Reviewer #1: I enjoyed reading the manuscript by Plenge and colleagues describing the establishment of an air-liquid interphase model of the porcine colonic epithelium. The introduction was well written and adequately presents the study rationale. The methods are appropriate for the study aim. The results are justified throughout the discussion, though the discussion may benefit from some refinement and focus. Overall this is an interesting study and presents a novel approach to allow future studies to experimentally assess the porcine colon without the need for harvesting fresh tissue.

Generalised comments:

1. Perhaps this is naivety as I work primarily with airway epithelium, but it is not clear why stem cells were utilised to construct 3D organoids prior to the 2D culture. Could the stem cells not have been directly differentiated in the ALI culture? The authors may wish to expand the rationale behind this step, as my understanding is that barrier function could still be assessed if proceeding directly to ALI culture.

- Thank you for your insightful question. We utilized stem cells to construct the 3D organoids prior to transitioning to the 2D culture. This approach was adopted because 3D organoids contain a higher proportion of stem cells, which are essential for generating the monolayer in the subsequent 2D culture. While direct differentiation of stem cells in the 3D culture is a viable option, it is not be suitable for the transport studies we intend to investigate. Additionally, the use of an air-liquid interface (ALI) culture was not employed in this study, as our focus was specifically on using a submerged 2D culture system to facilitate functional assays, such as electrophysiological measurements in Ussing chambers. ALI cultures are more commonly used to study airway epithelium. Therefore, we have opted to utilise the 2D culture. An alternative approach would be to use an undifferentiated 2D monolayer to form a new 2D monolayer. However, this would be less efficient in terms of both time and cost. The use of 3D organoids provides a more reliable and consistent source of stem cells, which is essential for the formation and differentiation of the monolayer.

2. The methods section overall is rather wordy. I think this could be significantly reduced to aid in readability – for example, the RNA extraction paragraph states the kit was performed according to manufacturer's instructions but then proceeds to describe each step.

- Thank you for your comments on the methods section. We have conducted a comprehensive rearranging of the section and agree that certain details could be streamlined to enhance readability. Therefore, the methods section has been significantly shortened to enhance readability (line 112).

3. The correct gene notation needs to be utilised throughout the manuscript.

- The gene notation has been verified throughout the manuscript and updated as required.

4. I am concerned about the small sample size used for many of the results (typically n=4). I do not think this necessary precludes publication, but I would strongly advocate for the graphs to be updated to include the individual data points so readers can better understand any variation present. The authors should also consider adding a line addressing that this study is a ‘proof of concept’ rather than a detailed comparison against native tissue.

- We are grateful for your considered response. We are aware of the issue of the small sample size and of the necessity to ensure that readers have a comprehensive understanding of the variability in our results. For the gene expression analysis, RNA was isolated from four independent 2D cultures of organoids, with each sample having two technical replicates to ensure the reliability and consistency of the data. Similarly, for the transport characteristics, four independent experiments were conducted, each with three technical replicates, thus facilitating an assessment of the reproducibility of the findings. This has also been included to the manuscript. It is also important to note that the organoids used in this study were derived from two pigs and the 3D organoid cultures were treated with high similarity to an immortalised cell line, thereby facilitating continuous passage and reproducibility due to their high amounts of stem cells. In this context, the passage of the 3D organoids used to generate the 2D cultures can be considered a biological replicate, thereby further reinforcing the robustness of the study. In accordance with your recommendation, we will incorporate individual data points into all the graphs, thus facilitating a more detailed visual representation of the data and any inherent variations.

In regard to the scope of the study, we acknowledge the necessity of explicitly delineating the context within which it is situated. Although this is an early-stage investigation, we have chosen not to frame the work as a proof of concept, as we believe this term can be somewhat limiting and often underestimates the value of studies that explore novel approaches. Instead, our intention is to position this work as a significant contribution towards a deeper understanding of the characteristics of organoid models, with the objective of stimulating further research in this area. We are committed to providing transparency regarding the experimental design, the limitations of the study, and the necessity for further validation in future studies. It is our intention that this approach will provide readers with a clear understanding of the study's intent and its position within the broader context of ongoing research in this field.

Specific comments:

5. Can the authors please provide additional information regarding the Animal Welfare Act that provides the ethics exemption (year and country).

- Thank you for your comment. We have included the information regarding the Animal Welfare Act in the document (line 71).

6. Very minimal information is provided in regard to the native tissue that was utilised. A brief sentence should be included in the methods regarding the RNA collection of native tissue – was it from the same animals or was this banked sample?

- Thank you for your feedback. The missing information has been included in the method section (line 109-110). The native tissue was sourced from different pigs from banked samples. The pigs were of the same breed and comparable age as the pigs which were used to isolated the crypts.

7. Lines 76 to 79 repeat the same information provided in lines 70-73.

- Thank you for bringing this to our attention, the repeated lines have been removed.

8. In Figure 1 it would be preferred if the actual TEER values were reported, rather than reporting a percentage. It is difficult to objectively determine whether a robust barrier was formed and compare to other studies when the results have been normalised to a previous timepoint.

- We appreciate your feedback regarding the reporting of TEER values. In the figure, we chose to report the relative TEER values as a percentage normalised to the day 8 as 100 %, as this approach allows for easier comparison of the relative changes in barrier integrity across time points. However, we understand that presenting the actual TEER values could offer additional clarity. To address this, we have included the mean TEER value on day 8 along with the standard deviation in the figure description. We hope this additional information will assist in evaluating the robustness of the barrier formation and facilitates comparisons with other studies.

9. Similarly, as mentioned earlier, figures 3-8 would significantly benefit from showing the individual data points on the graphs. Some of the error bars appear quite large and given the small number of samples it would help interpretation if individual data points are shown.

- Thank you for your comment. The figures 3-8 have been changed into discrete data points representing the results of the independent experiments.

10. The arrows in Figure 2 are a bit difficult to see. Could the authors consider enlarging the arrows and perhaps making them less transparent/brighter?

- Thank you for bringing this to our attention. The arrowheads have been modified to larger and, hopefully, brighter arrows to improve the visibility to the reader.

11. There is a minor typo in line 138 – should be epithelial.

- The typo has been fixed.

12. In line 140 and 141 the abbreviations Isc and Rt are used but a full definition is not provided.
13. The reference or references used in lines 188 and 189 appear to be corrupted/missing.
14. On line 223, the title should be shortened to ‘Ussing chamber studies show physiological transport capacity of 2D-organoids’ as the results do not present any native tissue to assess similarity.

- Thank you for your feedback; we have made the requested changes by providing full definitions for Isc and Rt, correcting the references on lines 137.

15. The discussion would benefit from a generalised introductory paragraph summarising the main findings. The authors may also wish to remove the subheadings from the discussion, as it could be argued that everything they assessed is considered barrier function.

- We are grateful for your valuable input. In response, we have incorporated a comprehensive introductory paragraph in the discussion section, summarising the primary findings (line 267 – 275). Additionally, we have eliminated the subheadings, aligning with your observation that all assessed elements pertain to the subject of barrier function.

16. The ‘Barrier Function’ component of the discussion could be streamlined – I can understand what the authors are trying to say and agree with the findings but it jumps between topics with little consistency. For example, Line 272 states “To gain further insight and utilise the model for the study of pathophysiology, an analysis of gene expression was conducted.” Which comes directly after a discussion on mucin gene expression and is immediately followed by discussion of TEER results. Similarly, a strength of the study is the comparison to native tissue, but the mucin similarity is not discussed. Yet it does discuss the comparative expression of the mucins, which one could argue is not particularly relevant if the mucin expression is the same as native tissue.

- We have refined the discussion to present a more logical and cohesive narrative.

17. Why do the authors speculate the Ussing chamber is not sensitive to carbachol induced changes when numerous other studies have used Ussing chambers to assess the effects of carbachol? Are they referring to the effect of this tissue specifically? Could it be a dosing issue? I am not familiar with the referenced papers assessing the carbachol response in native tissue, but were there experimental differences that could explain this?

- Thank you for your thoughtful comment and for raising an important point about the sensitivity of the Ussing chamber in our study. We would like to clarify that, based on our observations, we were specifically referring to the response of the tissue in our experimental conditions rather than making a broad statement about the sensitivity of the Ussing chamber. We acknowledge that numerous studies have successfully used the Ussing chamber to assess carbachol-induced changes, and in fact, many studies have employed the same concentration of carbachol that we used in our experiments (1, 2). This leads to the conclusion, that missing effects after carbachol application are not due to the methods sensitivity. Moreover, these findings may be explained by posttranslational modifications of the responsive transporter since the gene expression was highly comparable to the *in vivo* situation. We therefore removed parts of the text where the sensitivity of the method is discussed and criticized.

Reviewer #2: The work conducted by Plenge et al in this manuscript characterises a 2D porcine colonic organoid model for studying the physiology and barrier function of the gut. The manuscript is overall well-written, and the techniques used are appropriate. The authors were able to generate a 2D organoid model within 10 days of seeding that recapitulates many of the morphological and transcriptional characteristics observed in the native colonic epithelium, albeit with slight functional differences shown via Ussing chambers. This in vitro model could be utilised for translational research in gastrointestinal physiology, as well as testing intestinal-specific responses to therapeutic applications.

However, my major concern about the study pertains to the extremely limited sample size – the authors only had a biological replicate of two pigs. It is unclear whether the n numbers referenced in the figure legends are technical replicates per animal or the combined total over two animals, or whether multiple samples were taken from each pig. Nevertheless, such a small sample size does not provide the reader with any confidence that the mean (SD) is accurate. Primary cell cultures are known to display larger variations in their morphology and physiology compared to immortalised cell lines. Hence the authors should conduct additional studies to ensure their observations are indeed real and do not occur by chance.

- Thank you for your insightful comments and for highlighting the concern regarding the sample size. We recognize that the limited number of biological replicates may raise questions about the robustness and reliability of our findings. To clarify, the study used organoids isolated from two pigs, which were then passaged to generate the 2D cultures. Each of the four independent experimental conditions refers to a biological replicate generated from a separate passage of 3D organoids, with each passage used to create a separate 2D culture. Each biological replicate was subjected to two technical replicates in the gene expression analysis and three technical replicates for the transport characteristics, as outlined in the figure legends. Respective changes in the manuscript have been made.

We understand that primary cell cultures can exhibit more variability compared to immortalized cell lines, and we are aware of the potential for variation in such models. However, despite the small sample size, the use of multiple technical replicates within each biological replicate helps to control for variability and provide more reliable data. Furthermore, we agree that additional studies with larger sample sizes are necessary to validate these initial findings. We are committed to expanding this research to further investigate the observed trends and to provide more robust data for future publication.

In response to your feedback, the figure legends have been updated to provide greater clarity regarding the biological and technical replicates employed in the study. We hope that this additional context will assist in addressing your concerns regarding the sample size and variability.

There are a few grammatical and typographical errors that should be amended before acceptance in the journal. The authors are referred to the uploaded feedback where a number of these errors (but not exhaustive) are highlighted. Other general comments are noted below:

1. Lines 75-79 are repeats of lines 70-73. The latter should be deleted.

- The repeated lines have been removed.

2. Line 80: Although the procedure has been described in Hoffman et al, the authors should still state which part of the intestine the samples were derived from. Is it also the jejunum? Additional description would be helpful.

- Thank you for your comment, in order to provide further clarification, we have amended the methodology section to include a description of the specific segment of the intestine used to create the organoids (line 76-77).

3. Line 82: what medium? The reference to S1 table should be made here. Clarify as 'organoid medium' (S1 Table).

4. Line 86: Again, authors are advised to specify which medium - organoid media

- We have clarified which media was used and added the reference to the S1 table.

5. Line 125-6: genes should be italicised

- The relevant genes have been italicised.

6. Line 138: unclear how many technical replicates were performed for the Ussing chambers? Is it at least 3?

- To ensure the reliability of the data, we performed at least three technical replicates for the Ussing chamber experiments. We have also added a sentence in the Methods section to provide clearer understanding (line 149-150).

7. Line 140: First time the acronym is used - should be spelled out here. Short-circuit current (Isc) and resistance (Rt)

- The acronyms have been spelled out in accordance with the relevant guidelines.

8. Line 142: Is Table 1 also the order that the substances were tested?

9. Line 142-3: If all cultures had aldosteroine pre-incubation, the highlighted sentence should immediately follow the first sentence after reference to Hoffman et al.

- The table does not indicate the order in which the substances were tested. Each substance was tested in a separate setup, followed by the application of forskolin as a viability test. For the aldosterone testing, an additional substance was included. Only the cultures tested with aldosterone were pre-incubated with various aldosterone concentrations. After pre-incubation, the membranes were mounted in the Ussing chamber, and amiloride was applied before forskolin. To provide clearer understanding, the methods section has been rephrased (line 139-141).

10. Technically, it would have been nice to see the native tissue also mounted on the Ussing chamber as a true control to the 2D organoids to compare functional physiology.

- While we appreciate the suggestion, mounting native tissue on the Ussing chamber as a direct control to the 2D organoids was not included in this study. The functional physiology of native tissue in the Ussing chamber has been extensively studied in previous research in our Institute (e.g. Leonhard-Marek et al. (1), Klinger et al. (3), Bridges et al. (4), Inagaki et al. (5)). Therefore, a direct functional comparison was not deemed necessary for the scope of this study.

11. Line 166: what mean values are being compared? Also the same for line 168 – what is the measured variable being compared?

- The text has been revised to specify that the mean values of gene expression are being compared (previously stated in line 166, now line 167). Additionally, it has been clarified that the comparison involves gene expression across different cultivation times (previously stated in line 168, still line 168).

12. Line 172: I disagree with the statement “the number of biological experiments”. Please refer to comments above.

- We have addressed in a previous comment, we clarified that the term refers to the number of independent replicates performed in our study.

13. Lines 176-178: arguably these sentences could be in the methodology section as it provides background information on the methodological approach.

- We respectfully disagree with the proposed relocation of these sentences to the methodology section. They represent a concise overview of the methodology pertinent to the ensuing discussion of results. The placement of these sentences in the results section provides context for the findings and facilitates a seamless integration of the methodology and observed outcomes.

14. Lines 188-9: Please fix Error Reference source not found

- The errors in the references have been rectified.

15. Line 190: Why not consider a simple H&E cross-section to complement the data? It would enable the visualization of the different cell types too. Although it is understandable to make assumptions working with monolayers.

- We agree that an H&E cross-section would be valuable for visualizing different cell types and tissue structure. In fact, we have previously performed H&E staining, and the data is published under Hoffmann (6). The results from this study confirmed the cellular composition and structure, which complement our current functional analysis.

16. Do the cultures continue to differentiate past day 10? What happens to the culture if you continue to maintain it?

- Thank you for your insightful question. Differentiation in our cultures is initiated on day 8, and it is crucial for maintaining culture integrity. If differentiation is not initiated by this time, we observe a significant drop in TEER, and the monolayer begins to detach. Continuing culture past day 10 is challenging, as the TEER declines at varying rates, and the monolayer often detaches. This is why we established day 10 as the endpoint for our study. We hypothesize that, without differentiation, the cultures face a spatial limitation around day 8 due to the high proliferative capacity of the stem cells, which may lead to overcrowding and instability in the monolayer.

17. Line 268: please italicise genes.

- The relevant genes have been italicised.

18. Line 327: Agree with the authors comments. Perhaps this reasoning can be expanded to the junctional proteins too -, it may be useful to observe with immunofluorescence whether the structural orientation of the proteins is the same as in native colon tissue. But limitations are well acknowledged.

- Thank you for your valuable comment. We agree that exploring the structural orientation of junctional proteins could provide further insights. To address this, we utilized electron microscopy to examine the villi and indicated the apical side of the epithelium, which allowed us to assess the overall orientation of the cells. Additionally, an H&E staining, as performed by Hoffmann (6) confirmed the presence of the characteristic single-layered, highly prismatic epithelium, which aligns with the expected structure of native colon tissue. This previous study was also referenced in the discussion to support our findings.

While immunofluorescence (IF) would indeed be an excellent approach to investigate the structural organization of junctional proteins, this study was focused on functional parameters rather than structural ones.

FIGURES
ALL figures: please show individual points instead of bar charts for transparency
1. Figure 1: authors are recommended to keep the y-axis origin as 0

- We would like to show the standard deviation (SD) in its entirety to provide a more detailed insight into the data. Keeping the y-axis origin as negative allows for a clearer representation of the variability and ensures that the full range of data is visible.

2. Figure 3: please show individual points instead of bar charts for transparency. Native is missing an ‘e’ at the end of the legend.

- Thank you very much, that has been done.

3. Figure 4: as above please show individual points. Missing error bars for day 9 of ZO-1 gene.

- Solved.

4. Figure 7: include aldosterone name on the x-axis for ease of understanding

- We have updated the figures to show individual points for greater transparency. The spelling error in the legend has been corrected, and the name "aldosterone" has been added under the x-axis for clarity.

# References

1.	Leonhard-Marek S, Hempe J, Schroeder B, Breves G. Electrophysiological characterization of chloride secretion across the jejunum and colon of pigs as affected by age and weaning. Journal of Comparative Physiology B. 2009;179(7):883-96.

2.	Pfannkuche H, Mauksch A, Gäbel G. Modulation of electrogenic transport processes in the porcine proximal colon by enteric neurotransmitters. Journal of Animal Physiology and Animal Nutrition. 2011;96(3):482-93.

3.	Klinger S, Lange P, Brandt E, Hustedt K, Schroder B, Breves G, Herrmann J. Degree of SGLT1 phosphorylation is associated with but does not determine segment-specific glucose transport features in the porcine small intestines. Physiol Rep. 2018;6(1).

4.	Bridges RJ, Rummel W, Simon B. Forskolin induced chloride secretion across the isolated mucosa of rat colon descendens. Naunyn-Schmiedeberg's Arch Pharmacol. 1983;323(4):355-60.

5.	Inagaki A, Yamaguchi S, Ishikawa T. Amiloride-sensitive epithelial Na+ channel currents in surface cells of rat rectal colon. Am J Physiol Cell Physiol. 2004;286(2):C380-90.

6.	Hoffmann P. Development of an in vitro model for studying physiological properties and pathogenicity mechanisms of gut diseases caused by zoonotic pathogens. Hannover: Stiftung Tierärztliche Hochschule Hannover; 2021.
