# Supplementary material for: Development and characterization of a 2D porcine colonic organoid model for studying intestinal physiology and barrier function
Source: PLoS One. 2025 May 7;20(5):e0312989. doi: 10.1371/journal.pone.0312989 (PMC12057940; doi:10.1371/journal.pone.0312989)
Supplement: Attachment — Submitted filename: rebuttal letter.docx [file pone.0312989.s009.docx]

**Review Comments to the Author**

Reviewer #1: The authors have done well to address the initial reviewer comments. I am satisfied with the science but have some minor comments to improve the manuscript:
The description of the native tissue has been added as requested; however, it is described without any prior introduction. For reader clarity, this should be slightly modified to make clear that native tissue was used for a comparator before they give the details of where the tissue was collected.

Thank you for your constructive comments on our manuscript. We have revised the methods section to introduce the native tissue as a comparator, providing an introduction.

The discussion would benefit from addressing the small sample size – it was rationalized in the reviewer responses but is not specifically addressed in the manuscript. The inclusion of specific sample sizes was an important and welcome addition. However, it is still a small sample and that does limit the study findings. Given many readers will be critical of the work because of the limited sample number, the authors should pre-emptively address these concerns.

Thank you for pointing this out. We have now addressed the small sample size directly in the manuscript. This should address concern from readers regarding this limitation.

Line 283: Space missing between mucins and were
Line 314: Typographical error in ‘expression’.
Line 330: Typographical error in ‘replicated’.

The typographical errors have been corrected.

Reviewer #2: Thank you to the authors for addressing many of the comments from the first review. The manuscript has improved clarity and transparency, with apt acknowledgment of model limitations and future investigations. This novel culture model will enable investigations into the pathophysiology of intestinal diseases, which are increasingly becoming prevalent.
Minor comments - Thank you for changing the figures to represent individual data points. However, error bars are missing from figures 3-8 (despite the figure legend indicating mean+- SD). While the individual datapoints allow readers to deduce the spread of the data, it would still be nice to have the T error bars to indicate the calculated SD. This can be added behind the datapoints.

Thank you for your positive feedback. We also appreciated your suggestion regarding the error bars. They have been added to the figures 3-8.
