# Supplementary material for: Development and characterization of a 2D porcine colonic organoid model for studying intestinal physiology and barrier function
Source: PLoS One. 2025 May 7;20(5):e0312989. doi: 10.1371/journal.pone.0312989 (PMC12057940; doi:10.1371/journal.pone.0312989)
Supplement: Attachment — Submitted filename: Response to Editor.docx [file pone.0312989.s010.docx]

Additional Editor Comments :

Dear Mrs Plenge,

We are currently reviewing your manuscript, titled “Development and Characterization of a 2D Porcine Colonic Organoid Model for Studying Intestinal Physiology and Barrier Function”.

During our assessment, we identified a highly similar manuscript published in Journal of Visualized Experiments titled “Two-dimensional Porcine Intestinal Organoids Reflecting the Physiological Properties of Native Gut, DOI: 10.3791/67666.” The significant overlap in content between the two manuscripts raises concerns regarding potential duplication of publication.

To ensure transparency and adherence to ethical publishing standards, we kindly request clarification on the following points:

1) The relationship between the two manuscripts (e.g., whether they represent distinct studies or overlapping analyses).

2) Whether the submission to Journal of Visualized Experiments was made with the understanding that the content had already been accepted/published elsewhere.

3) Any additional context or justification for the similarities between the manuscripts.

We take issues of publication ethics very seriously and aim to resolve this matter promptly. Your response will help us better understand the situation and determine the appropriate next steps.

Please provide your clarification at your earliest convenience. If you have any questions or require further assistance, do not hesitate to reach out to the PLOS One team.

Thank you for your attention to this matter, and we look forward to your response.

Dear Editor,

Thank you for your detailed feedback regarding the perceived overlap between our revised manuscript and our previously published work in the Journal of Visualized Experiments (DOI: 10.3791/67666). We appreciate the opportunity to clarify the relationship between these two studies.

1. Independence of the studies

Although both manuscripts use the two-dimensional porcine intestinal organoid model, they are based on independent experiments with different scientific objectives. The JOVE paper focuses primarily on establishing and visually demonstrating the protocol for generating 2D organoid cultures, thus providing a validated methodological framework. In contrast, our revised manuscript presents a detailed functional and molecular characterisation of the model, with particular emphasis on intestinal barrier integrity, ion transport and gene expression.

2. Experimental use of different donor animals and timeline

The two studies were performed using organoids derived from different donor animals, as reflected by the different animal registration numbers reported in the respective manuscripts (e.g. JOVE: TiHo-T-2023-15; revised manuscript: TiHo-T-2017-22 and TiHo-T-2024-5). In addition, the experiments were carried out in different time periods, further confirming their independence. Despite these differences, the high similarity of the results obtained in both studies underlines the robustness and reproducibility of the 2D porcine organoid model.

3. Justification for Methodological Overlap

As both studies use the same well-established organoid system, it is inevitable that certain parts of the materials and methods will be similar. However, the research questions, experimental approaches and data analyses are distinctly different between the two manuscripts. The JOVE study serves as a methodological reference, while the revised manuscript extends this work by exploring unique aspects of the model that are critical for understanding its physiological and pathophysiological properties.

In summary, although both manuscripts share a common experimental platform, they represent independent investigations with complementary but non-duplicative aims. We hope that this clarification reinforces our commitment to transparency and adherence to ethical publishing standards.

Sincerely,

Masina Plenge
